# HDAC3 But not HDAC2 Mediates Visual Experience-Dependent Radial Glia Proliferation in the Developing *Xenopus* Tectum

**DOI:** 10.3389/fncel.2016.00221

**Published:** 2016-09-27

**Authors:** Juanmei Gao, Hangze Ruan, Xianjie Qi, Yi Tao, Xia Guo, Wanhua Shen

**Affiliations:** ^1^Zhejiang Key Laboratory of Organ Development and Regeneration, College of Life and Environmental Sciences, Hangzhou Normal UniversityHangzhou, Zhejiang, China; ^2^Department of Neurosurgery, Nanjing Medical University and Jiangsu Cancer HospitalNanjing, Jiangsu, China

**Keywords:** histone deacetylase, radial glia, proliferation, *Xenopus laevis*, visual experience

## Abstract

Radial glial cells (RGs) are one of the important progenitor cells that can differentiate into neurons or glia to form functional neural circuits in the developing central nervous system (CNS). Histone deacetylases (HDACs) has been associated with visual activity dependent changes in BrdU-positive progenitor cells in the developing brain. We previously have shown that HDAC1 is involved in the experience-dependent proliferation of RGs. However, it is less clear whether two other members of class I HDACs, HDAC2 and HDAC3, are involved in the regulation of radial glia proliferation. Here, we reported that HDAC2 and HDAC3 expression were developmentally regulated in tectal cells, especially in the ventricular layer of the BLBP-positive RGs. Pharmacological blockade using an inhibitor of class I HDACs, MS-275, decreased the number of BrdU-positive dividing progenitor cells. Specific knockdown of HDAC3 but not HDAC2 decreased the number of BrdU- and BLBP-labeled cells, suggesting that the proliferation of radial glia was selectively mediated by HDAC3. Visual deprivation induced selective augmentation of histone H4 acetylation at lysine 16 in BLBP-positive cells. Furthermore, the visual deprivation-induced increase in BrdU-positive cells was partially blocked by HDAC3 downregulation but not by HDAC2 knockdown at stage 49 tadpoles. These data revealed a specific role of HDAC3 in experience-dependent radial glia proliferation during the development of *Xenopus* tectum.

## Introduction

The neural cells, including neurons and glia are generated from progenitor cells and functionally incorporated into neural circuits during the maturation of central nervous system (CNS; Raymond and Easter, 1983; Ruthazer and Cline, 2004; Kaslin et al., 2008; Kriegstein and Alvarez-Buylla, 2009; Sild and Ruthazer, 2011). It is known that stem cells are the main sources for the proliferation and differentiation of progenitor cells (Kriegstein and Alvarez-Buylla, 2009). Radial glial cells (RGs) have been shown as one of the major forms of progenitor cells in human (Kipp et al., 2011), rat (Hartfuss et al., 2001), *Xenopus* (Tremblay et al., 2009; Sharma and Cline, 2010; D’Amico et al., 2011; Bestman et al., 2012; Guo et al., 2015; Tao et al., 2015) and zebrafish (Ito et al., 2010). They can also act as scaffolds for the migration of newly generated neurons (Costa et al., 2010) and guide the retinal ganglion cells in projecting retinal axons to form functional topographic mapping in the tectum (Braisted et al., 1997; Schmitt et al., 2006). RGs are easily identified by their distinctive profiles with single elongated processes, periventricular cell bodies and expanded end feet (Kriegstein and Alvarez-Buylla, 2009; Tao et al., 2015). In the *Xenopus* brain, most RGs are progenitor cells that distributed along the ventricular layer in optic tectum (Sharma and Cline, 2010; Bestman et al., 2012; Tao et al., 2015). RGCs are beginning to differentiate into neurons and glia at stage 39 tectum (Wu et al., 1999). Visual activity dynamically regulates the newly generated cells by RGs in the developing retinotectal circuit (Sharma and Cline, 2010; Tao et al., 2015).

Regulation of the proliferation of progenitor cells is multiplied by a variety of factors, such as neural trophic factors (Zhao et al., 2008), neurotransmitters (Deisseroth et al., 2004) and electrical activity (Spitzer, 2006). Our previous studies showed that histone deacetylase 1 (HDAC1) regulates the activity-dependent proliferation of RGs in the developing *Xenopus* tectum (Tao et al., 2015). The highly conserved class I HDACs consists of four superfamily members (HDAC1, HDAC2, HDAC3 and HDAC8; Haberland et al., 2009). However, whether other HDAC members are also involved in the regulation of RG proliferation during brain development remains unknown. For this study, we investigated whether sensory experience-dependent proliferation of RGs is regulated by HDAC2 or HDAC3 in the developing optic tectum of *Xenopus* *laevis*.

The tadpoles undergo high neurogenesis and regenerative capacities at the early stage brain (Bernardini et al., 2010). Optic tectal neurons receive the projections from retinal ganglion cells to function as a visual information processing center, which guide the visual avoidance behavior from stages 46 to 49 in response to visual stimulation (Dong et al., 2009; Shen et al., 2011, 2014). Visual experience has been shown to promote neuronal dendritic arbor growth (Sin et al., 2002) and increase the integration of neurons into neural circuits (Aizenman and Cline, 2007). Visual stimuli decreases the proliferation rate (Sharma and Cline, 2010), whereas visual deprivation increases the proliferation of RGs (Tao et al., 2015). Radial glia can interact with tectal neurons to change the filopodial motility by cell calcium transients (Tremblay et al., 2009). Thus, understanding the process of RG proliferation offers important new insights into the mechanisms underlying the development of the brain.

Here, we reported that HDAC2 and HDAC3 expression were developmentally regulated during the tectal brain maturation. HDAC3 knockdown significantly decreased BrdU^+^ RGs. Visual deprivation-induced increase of radial glia proliferation was selectively blocked by HDAC3 knockdown but not by HDAC2 knockdown in the *Xenopus* tectum. These data revealed that different HDAC isoforms play distinct roles in RG proliferation and visual activity controls the radial glia proliferation by HDAC3 signaling in the developing brain.

## Materials and Methods

### Animals and Rearing

All operations for animals were performed according to the rules of the “Regulation for the Use of Experimental Animals in Zhejiang Province”. This study has been approved by the local ethics committee of Hangzhou Normal University. Homebred tadpoles were obtained by mating male and female adult *albino* *Xenopus* injected with human chorionic gonadotropin (HCG). All tadpoles were reared in Steinberg’s solution [(in mM): 10 HEPES, 58 NaCl, 0.67 KCl, 0.34 Ca(NO_3_)_2_, 0.83 MgSO_4_, pH 7.4] within a 20°C incubator on a 12 h dark/light cycle. Tadpoles were anesthetized in 0.02% MS-222 (3-aminobenzoic acid ethyl ester methanesulfonate, Sigma-Aldrich) for experimental manipulations. Stages of tadpoles were characterized according to the developmental changes in the anatomy (Nieuwkoop and Faber, 1956). For visual deprivation, tadpoles were placed in a black plastic box at a 20°C incubated for 48 h.

### Immunohistochemistry

Tadpoles were anesthetized in 0.02% MS-222, and fixed in 4% paraformaldehyde (PFA, pH 7.4) at 4°C overnight or room temperature for 2 h. Tadpoles were rinsed with 0.1 M PB and immersed in 30% sucrose overnight for dehydration. Animals were embedded in optimal cutting temperature (OCT) media and cut into 20 μm cryostat sections with a microtome (Microm, HM550 VP). Sections were rinsed with 0.1 M PB for 2 × 20 min, and permeabilized with 0.3% Triton X-100 in PB, and blocked in 5% goat serum for 1 h before incubating with primary antibodies at 4°C overnight. For primary antibodies, we used the antibodies of SOX2 (1:100, Rabbit, ab97959, Abcam), HDAC2 (1:200, Rabbit, ab137364, Abcam), HDAC3 (1:200, Rabbit, ab16047, Abcam), H2BK5Ac (1:600, Rabbit, ab40886, Abcam), H4K5Ac (1:600, Rabbit, ab51997, Abcam), H4K8Ac (1:600, Rabbit, ab45116, Abcam), H4K12Ac (1:600, Rabbit, ab46983, Abcam), H4K16Ac (1:600, Rabbit, ab109463, Abcam), H3K9Ac (1:600, Rabbit, ab10812, Abcam), and BLBP (1:200, Rabbit ab324223, or Mouse, ab131137, Abcam). Sections were rinsed with PB and incubated with the secondary antibody (FITC or Rhod) for 1 h at room temperature. After sections were counterstained with DAPI, mounted on slides and sealed with clear nail polish, the immunofluorescent images were collected using a confocal microscope (LSM710, Zeiss, Germany).

### Western Blot

Tadpoles were anesthetized in 0.02% MS-222. The optical tectum was exposed by peeling off the wrapped skin and dissected. The tecta (about 10 ~ 20 brains for each group) were homogenized in the radioimmunoprecipitation assay (RIPA) buffer with a protease inhibitor cocktail (1:100, Sigma Aldrich) at 4°C. Protein homogenates were measured by BCA assay using a Nanodrop (Thermo Scientific, 2000c), separated by SDS-PAGE (10%, Bio-Rad) and transferred to PVDF membranes. Membranes were blocked in 4% nonfat milk containing 0.1% Tween-20 (Sigma Aldrich; TBST) for 1 h at room temperature and incubated with primary antibodies overnight at 4°C. Antibodies of HDAC2 (1:1000, ab137364, Abcam), HDAC3 (1:1000, ab16047, Abcam), GAPDH (1:5000, GR68497–2, Millipore) were diluted in 4% nonfat milk. Blots were rinsed with TBST and incubated with horseradish peroxidase (HRP)-conjugated secondary antibodies (1:2000, Invitrogen) for 1 h at room temperature. Bands were visualized using ECL Chemiluminescent Substrate Kit (Pierce).

### BrdU Labeling and Image Analysis

BrdU labeling was modified according to the previous studies (Tao et al., 2015). The tadpoles were incubated with 5-bromo-2-deoxyuridine (BrdU, 10 mM, MP Biomedicals, Solon, OH, USA) in Steinberg’s solution for 2 h and anesthetized for fixation. Brain was sectioned and treated with 2 N HCl for 45 min at 37°C to denature the DNA. The sections were rinsed with 0.1 M PB, washed three times with PB containing 0.3% Triton X-100 and incubated in 5% normal goat serum in PB for 30 min. The sections were incubated with a BrdU monoclonal primary antibody (1:100, Sigma) overnight at 4°C. BrdU-labeled S phase nuclei were visualized by incubation with FITC- or Rhodamine-conjugated goat anti-mouse secondary antibody.

For BrdU and BLBP counting, eight representative sections of each tectum were collected for analysis. The first section was taken, where the two tectal lobes meet at the midline of ventricular layer and the last section was taken where the anterior ventricle appears at the midline. The brain sections were scanned by a confocal microscopy and analyzed by an image processing software (iMaris, Surphaces module, Bitplane AG, Zürich, Switzerland). For each section, the region selected for cell number counting was delineated by the anterior commissure to the caudal curvature onset on one axis and the midline to the neuropil side (20 μm) on the perpendicular axis. The number of BrdU and BLBP labeling cells from all eight sections were added counted as the total cells from one brain.

For enhanced acetylation fluorescent cells counting, five consecutive sections of each tectum were selected for analysis. The fluorescent images were converted to monochrome images and the background fluorescent intensities were measured from the entire cell layer within the tectum. The acetylated cells with two-fold higher intensities than the background intensities were selected and counted. The region for BrdU^+^ cells counting were used for comparison.

### Morpholinos and Tectal Cell Transfection

To knock down the endogenous HDAC2 or HDAC3 expression, we used translation-blocking morpholinos (MOs) against the *Xenopus* HDAC2 (HDAC2-MO, TATACGCCATGAAGAGCCCTGGAAC) or* Xenopus* HDAC3 (HDAC3-MO, TCTTTGCCATTTCGCTGCCACAGAC, GeneTools, Philomath, OR, USA). The control MO (Ctrl-MO, GATGGCATGTCTCCTCGCCTTTGGA) was also synthesized by Gene Tools Company. All morpholinos were tagged with Lissamine for fluorescent visualization. For whole brain electroporation, tadpoles at stage 45 were anesthetized and morpholinos (10 μM) were injected into the midbrain ventricle. The two paralleled platinum electrodes were placed on the skin above the tectum and current pulses were applied by a stimulator (Haas et al., 2002). The tadpoles were screened and only highly transfected tadpoles were used for further experiments.

### Statistics

Paired data were tested with Student’s *t*-test. Multiple group data were tested with an ANOVA followed by *post hoc* Tukey’s test unless noted. Data are represented as mean ± SEM. Experiments and analysis were performed blind to the experimental condition unless noted.

## Results

### Characterization of Radial Glial Cells and Sox2-Positive Progenitor Cells in The Developing *Xenopus* Tectum

**Figure 1:**
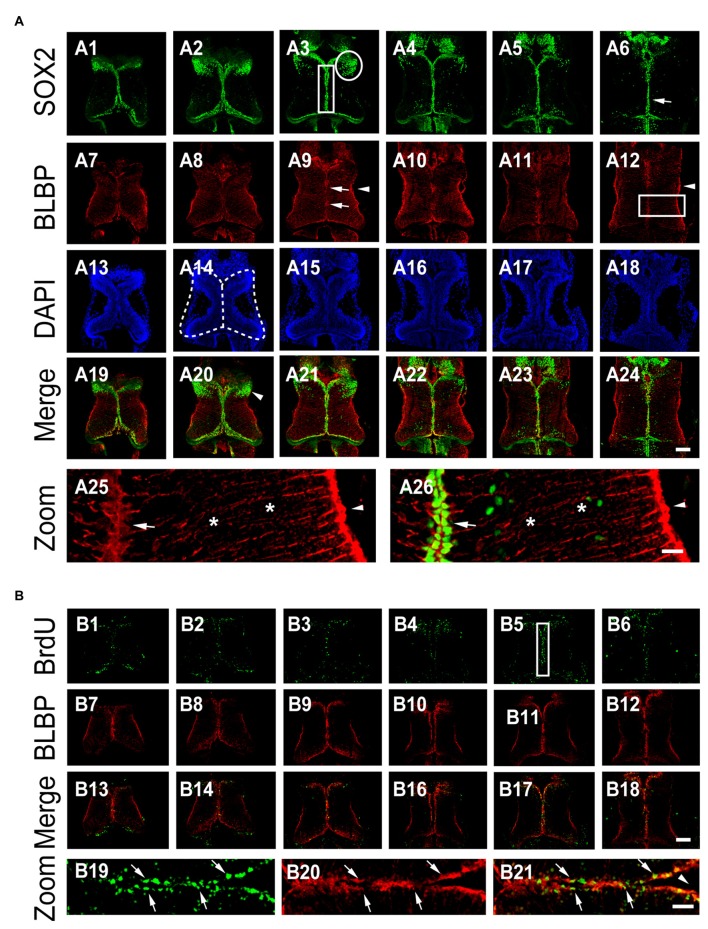
**BLBP^+^ radial glia are SOX2^+^ cells in the *Xenopus* tectum. (A)** Optic tectum were co-labeled with anti-SOX2 and anti-BLBP antibodies at stage 48. Six representative sections of the whole tectum were shown (A1–A6: SOX2; A7-A12: BLBP; A13–A18: DAPI and A19–A24: SOX2 and BLBP merged). Arrow heads indicate the endfeet of Radial glial cells (RGs; A12, A25, and A26); Arrows indicate cells stained for DAPI and SOX2; Stars indicate the processes of radial glia; White circle indicates the cluster of SOX2^+^ cells (A3); White square indicates the area for counting the number of BrdU^+^ cells (A3). Stars indicate the elongated processes of RGs (A25-A26). Scale: 100 μm (zoom in: 20 μm). **(B)** Tadpoles were incubated with BrdU for 2 h and co-labeled with anti-BrdU and anti-BLBP antibodies at stage 47. Six representative sections were shown (B1–B6: BrdU staining; B7–B12: BLBP staining and B13–B18: BrdU and BLBP merged). White square (B5) indicates the area for the zoom in images (B19–B21). Scale: 100 μm (zoom in: 20 μm).

To determine the location of neural progenitor cells (NPCs) in the optic tectum *in vivo*, we used an antibody of NPC marker sex-determining region Y-box 2 (SOX2) to immunostain the whole tectum at stage 48 (Figure 1A). Immunofluorescent images from the whole brain sections were scanned with a confocal microscope. We found that most SOX2-immuno positive (SOX2^+^) NPCs were present along the ventricle of the optic tectum (Figures 1A1–A6). A cluster of SOX2^+^ NPCs was distributed in the anterior forebrain of the optic tectum (Figure 1A3, circle). To determine whether SOX2^+^ cells are RGs, we immunostained the tectum with an anti-BLBP antibody, a well-characterized marker of RGs (Feng et al., 1994; D’Amico et al., 2011; Figures 1A7–A12). We observed that BLBP-labeled RGs have characteristic triangular cell bodies and single elongated processes with swollen endfeet that extended into the lateral neuropil (Figure 1A25). The BLBP^+^ RGs were only colocalized to those SOX2^+^ cell bodies which were distributed along the midline of the ventricle (Figure 1A26), indicating that RGs are NPCs at stage 48 tectum.

To determine whether BLBP^+^ RGs are dividing NPCs, we evaluated BrdU incorporation by exposing tadpoles to BrdU (10 mM) for 2 h (see “Materials and Methods” Section for details; Peunova et al., 2001; Sharma and Cline, 2010; McKeown et al., 2013; Tao et al., 2015). The tectal brains were immunostained with anti-BrdU and anti-BLBP antibodies at stage 48 (Figure 1B). BrdU^+^ or BLBP^+^ cells were counted from the whole tectum with an iMaris software (Figure 1B). We found that most of the BrdU-labeled cells were BLBP^+^ RGs at stage 48 (~80.6%), indicating that dividing progenitor cells were RGs (Figure 1B). These data suggest that most of periventricular BrdU^+^ RGs were dividing proliferative cells in the developing tectum. We evaluated the number of BrdU^+^ and BLBP^+^ RGs located along the ventricle of the tectum for the changes in cell proliferation (Figure 1B6).

### Class I HDAC Activity is Required for Radial Glial Cell Proliferation

**Figure 2:**
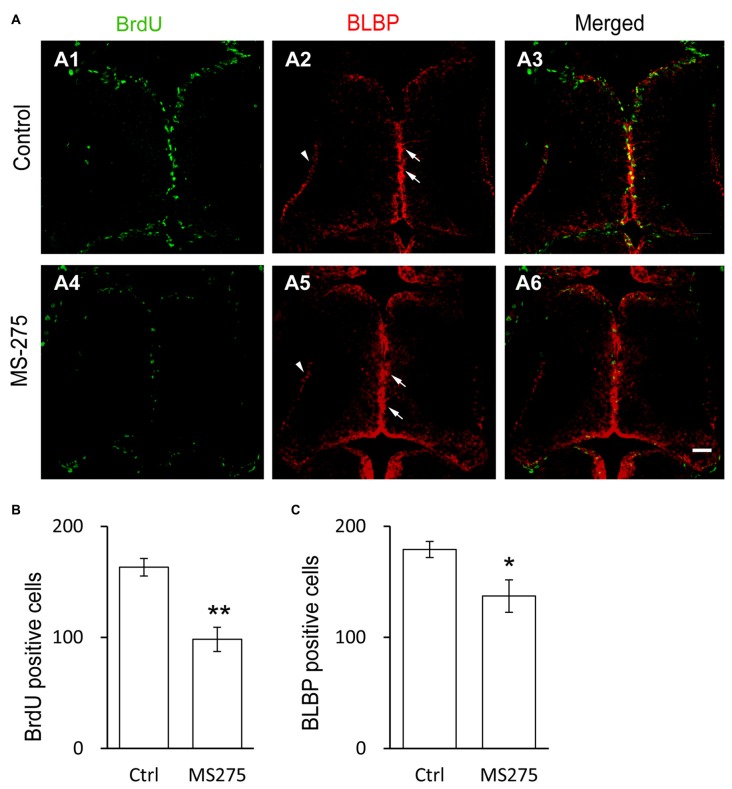
**Class I HDAC inhibitor decreases the proliferative rate of radial glia cells. (A)** Representative images showing the co-labeling for BrdU^+^ and BLBP^+^ in control (A1–A3) and MS-275-treated (10 μM, A4–A6) tecta. The BLBP^+^ cell bodies locate along the midline of the ventricular layer of the tectum (arrows) and the endfeet reside along the edge of neuropil (arrow heads). Scale: 50 μm. **(B–C)** Summary data showing that the numbers of BrdU^+^ and BLBP^+^ cells were dramatically decreased in MS-275-treated tecta compared to the control tecta. (BrdU: Ctrl, 163.2 ± 7.9, *N* = 5, MS-275, 98.2 ± 10.9, *N* = 4; BLBP: Ctrl, 179.2 ± 7.2, *N* = 5, MS-275, 137.2 ± 14.6, *N* = 4; *p < 0.05, **p < 0.01).

To test whether class I HDACs was involved in RG proliferation in the optic tectum, tadpoles at stage 46 were exposed to Entinostat (MS-275, 10 μM, Selleck), a class I HDACs inhibitor (Hu et al., 2003; Bahari-Javan et al., 2012) in Steinberg’s solution for 48 h. The tadpoles were incubated with BrdU for 2 h and immunostained with anti-BrdU and anti-BLBP antibodies (Figure 2A). We observed that the numbers of BrdU^+^ and BLBP^+^ RGs were dramatically reduced in the MS-275-treated tadpoles (Figures 2B,C). These data indicated that class I HDAC activity was required for the proliferation of RGs.

### Developmental Regulation of HDAC2 and HDAC3 Expression in Radial Glial Cells

**Figure 3:**
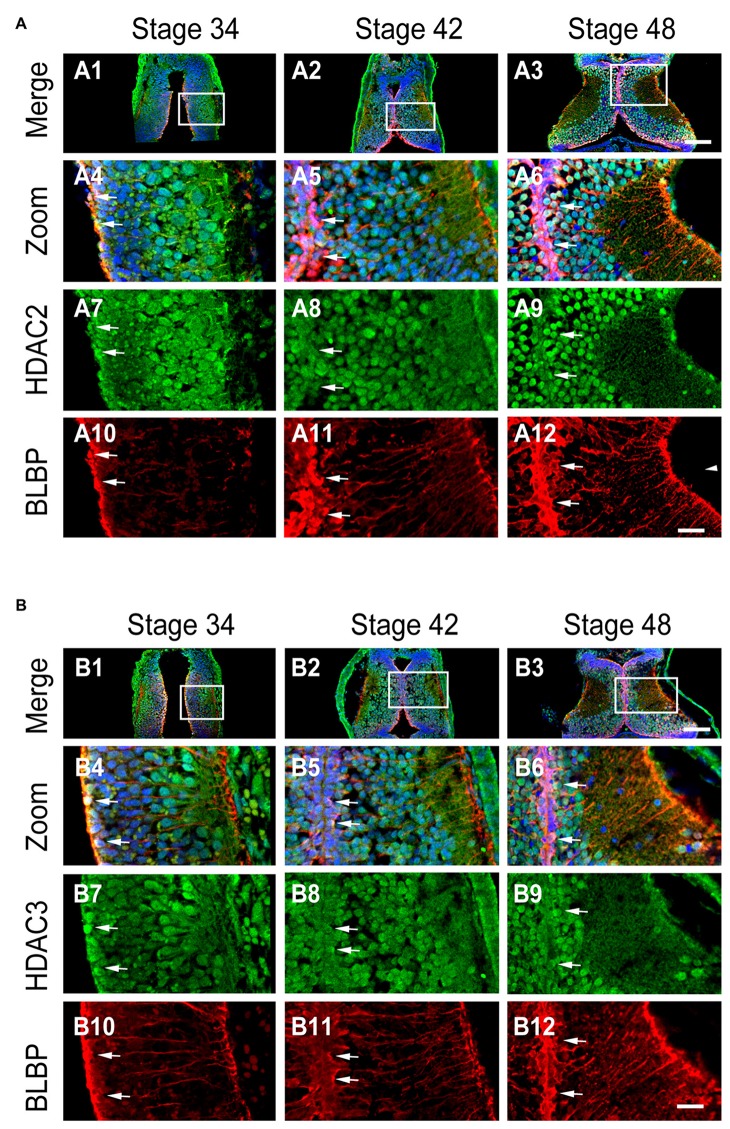
**Developmental changes in HDAC2 or HDAC3 and colocalization with BLBP in the optic tectum. (A)** Representative images showing the colocalization of HDAC2 and BLBP immunostaining at stages 34 (A1, zoom in: A4, A7, A10), 42 (A2, zoom in: A5, A8, A11), and 46 (A3, zoom in: A6, A9, A12) respectively. Arrow heads indicate the BLBP^+^ RGs processes. Arrows indicate BLBP^+^ RGs that are HDAC2^+^. Scale: 100 μm (zoom in: 20 μm). **(B)** Representative immunofluorescent images showing colocalization of HDAC3 and BLBP staining at stages 34 (B1, zoom in: B4, B7, B10), 42 (B2, zoom in: B5, B8, B11), and 48 (B3, zoom in: B6, B9, B12) respectively. Arrows indicate HDAC3 containing BLBP^+^ RGs. Scale: 100 μm (zoom in: 20 μm).

Class I HDACs contain four family members (HDAC1, HDAC2, HDAC3 and HDAC8). HDAC1 is developmentally regulated at proliferative glial cells in murine brain (MacDonald and Roskams, 2008) and *Xenopus* tectum (Guo et al., 2015; Tao et al., 2015). To test whether HDAC2 and HDAC3 expression in RGs were changed with the brain maturation, we immunostained the tectum with the specific antibodies against HDAC2 and HDAC3 (Guo et al., 2015) in cryosections at stages 34, 42 and 48 (Figure 3A). We found that HDAC2 was transiently localized to the mitochondria as HDAC1 (Guo et al., 2015) at stage 34 (Figure 3A7), and mainly confined to the cell nuclei at stage 42 and 48 (Figures 3A8,A9).

To test whether BLBP^+^ RGs contain HDAC2 or HDAC3, we co-labeled tectal cells with anti-BLBP (mouse) and anti-HDAC2 (Rabbit) or anti-HDAC3 (Rabbit) antibodies at stages 34, 42 and 48 (Figure 3B). We observed that most of BLBP^+^ cells along the ventricle layer of tectum expressed HDAC2 (Figure 3A) or HDAC3 (Figure 3B) at stage 34, 42 and 48 respectively. These data combined suggested that the subcellular localization of HDAC2 or HDAC3 was developmentally regulated and dividing progenitor cells expressed HDAC2 or HDAC3 in the tectum.

### HDAC3 But not HDAC2 Knockdown Decreases Cell Proliferation in the Optic Tectum

**Figure 4:**
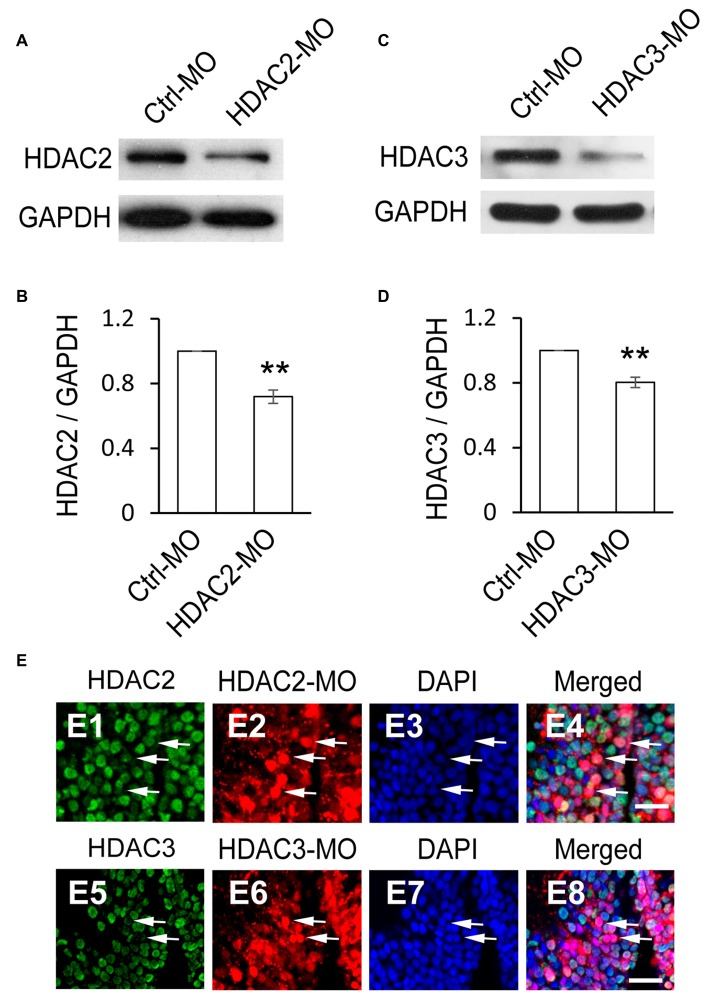
**HDAC2 or HDAC3 expression is decreased by HDAC2-MO or HDAC3-MO knockdown. (A)** Western blot analysis of homogenates from Ctrl-MO and HDAC2-MO transfected brains using an anti-HDAC2 antibody. **(B)** Quantification revealed that HDAC2 expression was significantly decreased in the HDAC2-MO transfected tectum compared to controls. **(C)** Ctrl-MO and HDAC3-MO transfected brain homogenates were compared using an anti-HDAC3 antibody.** (D)** HDAC3 expression was significantly decreased in the HDAC3-MO tectum. Data is represented as an intensity ratio of HDAC2 or HDAC3 to GAPDH normalized to the control value. Two-tailed *T*-test, *N* = 3, **p < 0.01. **(E)** Representative immunofluorescent staining of HDAC2 at HDAC2-MO-transfected cells (E1–E4) and HDAC3 at HDAC3-MO-transfected cells (E5–E8) in stage 48 tadpoles. Arrows indicate HDAC2/3-MO-transfected RGs stained for HDAC2/3. Scale: 20 μm.

**Figure 5:**
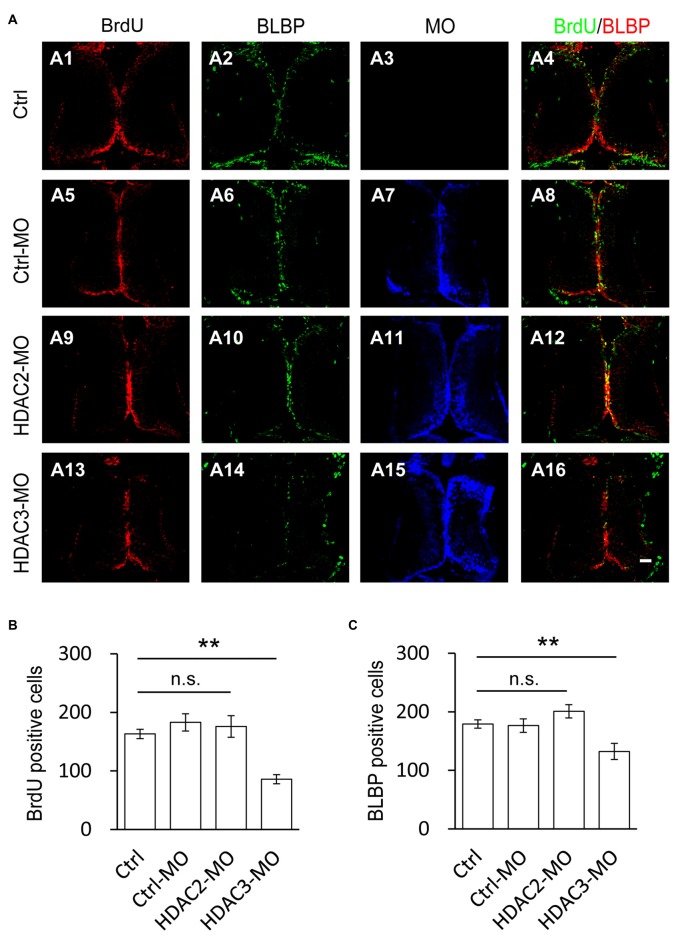
**Number of BrdU^+^ cells is decreased by HDAC3 knockdown but not HDAC2 knockdown. (A)** Representative immunofluorescence images of BrdU- and BLBP-labeled cells in control (A1–A4), Ctrl-MO (A5–A8), HDA2-MO (A9–A12) and HDAC3-MO (D13–D15) transfected brains in stage 48 tadpoles. Scale: 50 μm. **(B–C)** Summary data showing that HDAC3-MO, but not HDAC2-MO transfection dramatically decreased the number of BrdU- **(B)** and BLBP^+^ labeled cells **(C)**. It was not significantly altered in Ctrl-MO tectum ** (B,C)**. (BrdU: Ctrl, 163.2 ± 7.9, *N* = 5, Ctrl-MO, 183.0 ± 14.6, *N* = 4, HDAC2-MO, 176.0 ± 18.6, *N* = 3, HDAC3-MO, 86.0 ± 7.8, *N* = 3; BLBP: Ctrl, 179.2 ± 7.2, *N* = 5, Ctrl-MO, 176.5 ± 11.5, *N* = 4, HDAC2-MO, 201.0 ± 11.4, *N* = 3, HDAC3-MO, 132.3 ± 13.8, *N* = 3; **p < 0.01).

To test whether HDAC2 or HDAC3 affected the proliferation of RGs, we used antisense morpholinos of HDAC2-MO or HDAC3-MO to downregulate each of the HDACs expression. Western blot analysis of brain homogenates demonstrated that HDAC2-MO injection in the tectum results in a 28.1% downregulation of endogenous HDAC2 (Figures 4A,B), whereas HDAC3-MO transfection results in a 19.7% knockdown of HDAC3 (Figures 4C,D). Immunofluorescent staining also indicated that HDAC2-MO or HDAC3-MO transfected cells showed less HDAC2 or HDAC3 expression (Figure 4E). Tadpoles were transfected with Ctrl-MO, HDAC2-MO or HDAC3-MO at stage 46 respectively and maintained in the Steinberg’s solution for 48 h (Figure 5A). Tadpoles at stage 48 were subjected to BrdU labeling and immunostained with the anti-BLBP and anti-BrdU antibodies for counting BrdU^+^ and BLBP^+^ cells (Figure 5A). Summary data showed that the numbers of BrdU^+^ cells were markedly reduced for the HDAC3-MO transfected tadpoles compared to untreated or Ctrl-MO controls (Figures 5A,B). The number of BLBP^+^ cells were also decreased in the HDAC3-MO brain tectum compared to control or Ctrl-MO brain (Figures 5A,C). However, the numbers of BrdU^+^ and BLBP^+^ cells in HDAC2-MO transfected tectum (Figure 5A) were not altered (Figures 5B,C). These data suggested that HDAC3 but not HDAC2 knockdown reduced the number of BrdU^+^ and BLBP^+^ RGs in the tectum.

### Visual Deprivation-Induced Selective Elevation of H4K16 Acetylation

**Figure 6:**
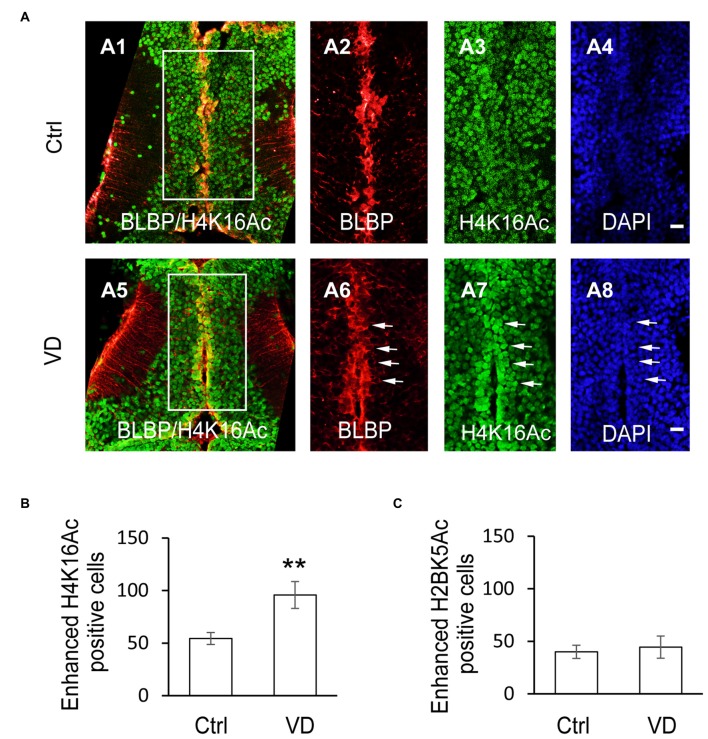
**Visual experience induces selective increase of histone H4 acetylation at lysine 16. (A)** Fluorescent images showing representative H4K16Ac^+^ cells in control (A1–A4), and VD (A5–A8) tadpoles. Scale: 50 μm. **(B)** Summary data revealed that visual deprivation increased the number of H4K16^+^ cells with enhanced fluorescence, which are colocalized with BLBP-labeled cells along the midline. **(C)** Summary data showing that VD barely changed the number of H2BK5^+^ cells with enhanced fluorescence. **p < 0.01.

To determine whether the histone acetylation was involved in the VD-induced increase of RG proliferation, tadpoles were exposed to VD and immunofluorescence was performed using the following antibodies: H2BK5Ac, H4K5Ac, H4K8Ac, H4K12Ac, H4K16Ac and H3K9Ac. Interestingly, VD stimulation significantly increased the number of enhanced fluorescent cells with acetylated H4K16 along the tectal midline, which are BLBP^+^ RGs (Figures 6A,B). The acetylation levels of H2BK5Ac (Figure 6C), H4K5Ac, H4K8Ac, H4K12Ac and H3K9Ac (data not shown) were not significantly changed following VD stimulation. These results indicated that VD stimulation induced a selective increase in the number of H4K16Ac^+^ RGs in *Xenopus* tadpoles.

### Visual Deprivation-Induced Increase in Radial Glial Cell Proliferation is Blocked by HDAC3 Knockdown But not By HDAC2 Knockdown

**Figure 7:**
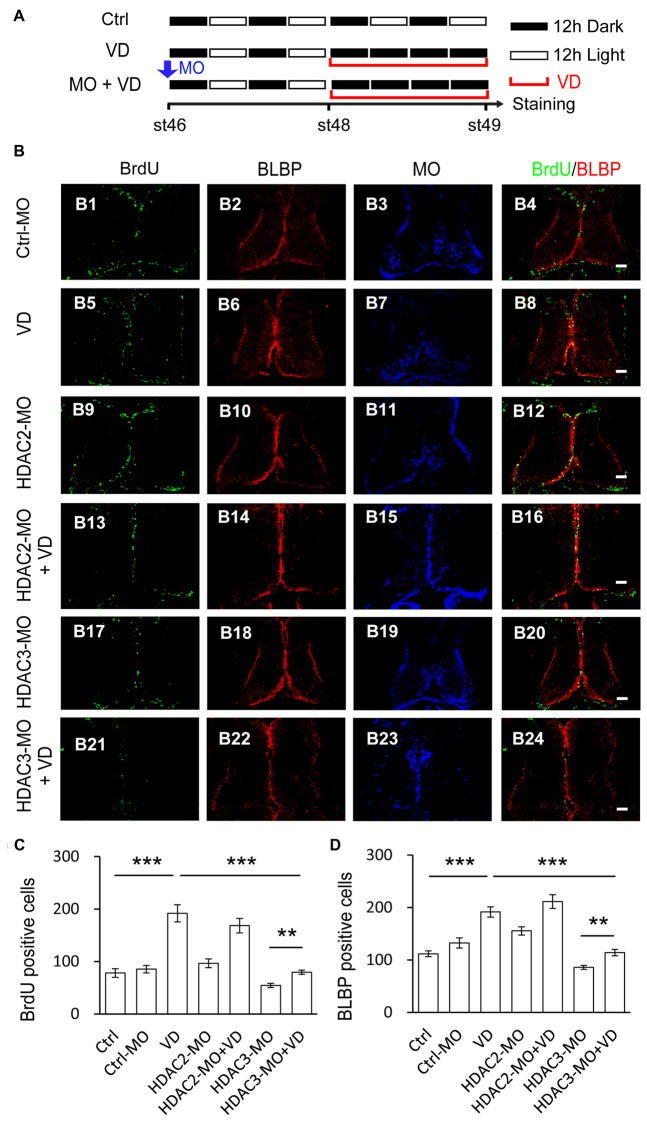
**Visual deprivation-induced increase of proliferative cells is prevented by HDAC3 knockdown but not HDAC2 knockdown. (A)** A cartoon showing that tadpoles at stage 46 were placed in a 12 h/12 h dark/light incubator for control, or a dark box for VD, or electroporated with HDAC2-MO/HDAC3-MO and placed in a dark box after 48 h for HDAC2-MO+VD/HDAC3-MO+VD. Tectal brains were labeled with BrdU at stage 49. **(B)** Representative fluorescent images showing BrdU- and BLBP-labeled cells in ctrl-MO (B1–B4), VD (B5–B8), HDAC2-MO (B9–B12), HDAC2-MO+VD (B13–B16), HDAC3-MO (B17–B20) and HDAC3-MO+VD (B21–B24) tadpoles. Scale: 50 μm.** (C–D)** Summary data showed that VD increased BrdU- and BLBP-labeled cells. HDAC3 but not HDAC2 knockdown partially blocked VD-induced increase of proliferative cells. (BrdU: Ctrl, 78.0 ± 8.3, *N* = 5, Ctrl-MO, 85.4 ± 7.1, *N* = 5, VD, 191.8 ± 16.4, *N* = 4, HDAC2-MO, 96.5 ± 8.5, *N* = 6, HDAC2-MO+VD, 168.2 ± 13.9, *N* = 4, HDAC3-MO, 54.2 ± 4.0, *N* = 4, HDAC3-MO+VD, 79.4 ± 4.1, *N* = 5; BLBP: Ctrl, 112.0 ± 5.6, *N* = 5, Ctrl-MO, 132.6 ± 9.7, *N* = 5, VD, 191.7 ± 9.9, *N* = 4, HDAC2-MO, 155.5 ± 7.8, *N* = 4, HDAC2-MO+VD, 211.5 ± 13.0, *N* = 5, HDAC3-MO, 86.0 ± 3.9, *N* = 4, HDAC3-MO+VD, 114.2 ± 6.0, *N* = 5; ***p < 0.001, **p < 0.01).

Visual deprivation (VD) is known to increase the proliferation of radial glia in optic tectum (Sharma and Cline, 2010; Tao et al., 2015). For visual deprivation, we placed tadpoles at stage 48 in a dark box for 48 h. Control tadpoles transfected with Ctrl-MO at stage 46 were maintained under the normal 12 h light/dark cycle until for BrdU incorporation. Animals were incubated with BrdU for immunostaining of anti-BrdU and anti-BLBP at stage 49 (Figure 7A). The number of BrdU^+^ and BLBP^+^ cells in the control tectum was dramatically increased in VD tadpoles compared to control tadpoles (Figures 7B–D). To further test whether other HDACs are HDAC2 and HDAC3 play roles in the VD-dependent RGs proliferation, we transfected tectal brains with HDAC2-MO or HDAC3-MO at stage 46. Tadpoles were maintained in a 12 h/12 h dark/light box for 48 h and exposed to darkness for 48 h. We observed that VD-induced increase of BrdU^+^ or BLBP^+^ cells was notably decreased in HDAC3-MO transfected tadpoles compared to control or Ctrl-MO tadpoles (Figures 7B–D). However, tadpoles transfected with HDAC2-MO did not alter the numbers of BrdU labeling of progenitor cells or block VD-induced increase in BrdU^+^ cells (Figure 7C). The number of BrdU^+^ cells was significantly decreased in HDAC3-MO transfected tadpoles compared to Ctrl or Ctrl-MO tadpoles. We also found that the number of BrdU^+^ cells was dramatically decreased in VD exposed HDAC3-MO tadpoles compared to VD exposed tadpoles, suggesting that HDAC3 knockdown partially blocks VD-induced increase of BrdU^+^ cells. Taken together, HDAC3 knockdown decreases the number of BrdU^+^ and BLBP^+^ cells at stages 48 tadpoles. HDAC3 is involved in VD-induced increase of cell proliferation (Figures 7B–D). These data suggested that HDAC3 activity is required for RGs proliferation in the developing tectum.

## Discussion

We showed that BLBP^+^ RGs are colocalized with SOX2^+^ progenitor cells along the ventricular layer of the optic tectum. Knockdown of HDAC3 but not HDAC2 dramatically decreased the number of BrdU^+^ cells along the midline of the tectum. Visual deprivation induced increase of BrdU^+^ cells was blocked by HDAC3 knockdown but not by HDAC2 knockdown. Taken together, we have demonstrated that HDAC3 selectively regulates the proliferation of RGs in the developing tectum of *Xenopus* *laevis* tadpoles.

NPCs are present in the restricted regions of developing brain. Radial glia maintain the pool of NPCs or neural stem cells (NSCs; Noctor et al., 2001; Merkle et al., 2004; Ogawa et al., 2005; Kriegstein and Alvarez-Buylla, 2009; Sild and Ruthazer, 2011), that differentiate into neurons or glia in the CNS (Noctor et al., 2001; Gregg and Weiss, 2003; Li et al., 2004; Gubert et al., 2009; Sharma and Cline, 2010; Bestman et al., 2012). Several stem cell markers are expressed in RGs, such as BLBP (fatty acid binding protein 7, Fabp7; Hartfuss et al., 2001; Anthony et al., 2004), SOX2 (Bestman et al., 2012), GFAP (Messenger and Warner, 1989) and vimentin (Tremblay et al., 2009). Vimentin or BLBP^+^ radial glia cells are one type of NPCs that reside in the ventricular layers in different model systems (Yoshida, 2001; Kiyota et al., 2008; Sharma and Cline, 2010; D’Amico et al., 2011, 2013; Tao et al., 2015). In the optic tectum, we found that BLBP^+^ RGs along the ventricular layer are also SOX2 immunoreactive. However, not all SOX2^+^ progenitor cells are RGs. One cluster of SOX2^+^ cells localized close to the rostral tectum are BLBP immuno-negative. The majority of BrdU^+^ dividing progenitor cells are RGs at the early stages of the *Xenopus* brain (Sharma and Cline, 2010; Tao et al., 2015). Tectal cells and migratory scaffold are mainly derived from RGs at late stages of developing chick tectum (Gray and Sanes, 1992).

The proliferation or differentiation of RGs are associated with a variety of factors (Peunova et al., 2001; Deisseroth et al., 2004; Spitzer, 2006; Mizutani et al., 2007; Del Bene et al., 2008; Zhao et al., 2008). HDAC activity are considered to be important for the proliferation of RGs. The proliferation of RGs was significantly decreased when animals were exposed to TSA, a broad inhibitor of class I/II HDACs (Almouzni et al., 1994; Tao et al., 2015). To specifically investigate the individual role of HDACs, many labs used knockout mice as a model to study their function. However, full HDAC1 knockout (Lagger et al., 2002) or HDAC3 mutant mice (Mano et al., 2014) are embryonic lethal. In the *Xenopus* system, we used morpholinos against HDACs to knockdown the protein expression in the developing *Xenopus* tectum *in vivo* to circumvent potential lethality concerns. Furthermore, tadpoles are easily manipulated for electroporation and *in vivo* observation. The whole brain immunohistochemistry also allows us to monitor the cell activity in the entire brain (Tao et al., 2015). We evaluated the proliferation rate of RGs by counting the number of BrdU^+^ and BLBP^+^ cells along the ventricular layer in the whole developing tectum. This technique in tadpoles provides us an ideal model to study the role of HDACs of the dynamic changes in cell proliferation in an intact brain *in vivo*.

It is traditionally thought that Class I HDACs (1, 2, 3 and 8) are mainly localized in cell nuclei (Abel and Zukin, 2008). HDAC1 and HDAC2 only have nuclear localization signal (NLS), while HDAC3 has both a nuclear export signal (NES) and NLS (Yang et al., 2002), suggesting that HDAC3 might localize to the cytoplasm (de Ruijter et al., 2003). HDAC3 is widely expressed in a variety of cells (Emiliani et al., 1998), including oligodendrocytes responsible for myelination (Shen et al., 2005; Broide et al., 2007) and striatum for Huntington disease (Gardian et al., 2005). HDAC3 activity is repressed during oligodendrocyte differentiation (Conway et al., 2012). In our experiments, HDAC2 and HDAC3 are almost localized to the nucleus at the stages from 42 to 48 that we examined. Most HDACs, including HDAC1 and HDAC3 are highly expressed in NSCs and reduced upon cell differentiation, except that HDAC2 is upregulated during neurons differentiation (MacDonald and Roskams, 2008; Montgomery et al., 2009). Interestingly, the distribution pattern between HDAC1 and HDAC3 at developmental tectum appears to be similar. Most HDACs participate in RG proliferation (Summers et al., 2013) by regulation of transcriptional repression at the development brain (Zupkovitz et al., 2006). HDAC1 (Tao et al., 2015) and HDAC3 but not HDAC2 knockdown significantly decreases BrdU- and BLBP-labeling cells at stage 48, indicating that HDACs selectively regulate the proliferation of RGs in the developing brain. This differential role of HDACs on cell proliferation might be the result of an associated deacetylase activity (de Ruijter et al., 2003).

The rate of radial glia proliferation is regulated by multiple intrinsic and extrinsic factors (Del Bene et al., 2008; Zhao et al., 2008; Costa et al., 2010; D’Amico et al., 2011; Dozawa et al., 2014). Visual experience plays an essential role in dendritic arbor structural plasticity and neural circuit development (Tao and Poo, 2005; Vislay-Meltzer et al., 2006; Shen et al., 2009; Xu et al., 2011). Visual activity is also one of the key factors that control the fate of radial glia proliferation during the brain development (Tremblay et al., 2009; Sharma and Cline, 2010; Bestman et al., 2012; Tao et al., 2015). Visual deprivation induced increases in radial glia proliferation is regulated by HDAC1 (Tao et al., 2015) and musashi1 (Sharma and Cline, 2010) in tadpoles. Radial glia can interact with retinotectal synapses and respond to visual stimuli to generate calcium transients (Tremblay et al., 2009), which provides a direct link that proliferation of RGs is modulated by visual activity. Overexpression of HDAC3 is selectively toxic for neurons through GSK3β phosphorylation of HDAC3 (Bardai and D’Mello, 2011), whereas loss of HDAC3 enhances long-term memory formation (McQuown and Wood, 2011). However, the potential roles of HDAC3 in RG proliferation is still unknown. We have shown that VD-induced increase of proliferative rate is selectively mediated by HDAC3 and HDAC1 (Tao et al., 2015). The regulation of HDAC activity might be through changing the acetylation level in tadpoles (Tao et al., 2015) and mice (Lagger et al., 2002). Although it is clear that epigenetic modulation is essential in RGs proliferation, more work is needed to determine the downstream signaling pathway by which HDAC3 selectively control the RG proliferation in the developing brain.

Our data clearly provide evidence that BLBP^+^ RG cells are SOX2^+^ progenitor cells. HDAC3 but not HDAC2 selectively down regulates BrdU^+^ RGs in the ventricular layer of the tectum. Furthermore, HDAC3 blocks visual deprivation-induced increase of BrdU^+^ progenitor cells. It will be important in the future studies to address the downstream signaling that control the proliferation and differentiation of radial glia for brain formation and repair.

## Author Contributions

All authors had full access to all the data in the study and take responsibility for the integrity of the data and the accuracy of the data analysis. JG, YT and WS: study concept and design. JG, HR, XQ, YT, XG and WS: acquisition of data. JG and WS: analysis and interpretation of data. WS: drafting of the manuscript. JG, HR and WS: statistical analysis.

## Conflict of Interest Statement

The authors declare that the research was conducted in the absence of any commercial or financial relationships that could be construed as a potential conflict of interest.
